# Graduate Study in Scotland and Ireland

**Published:** 1911-09-02

**Authors:** 


### GRADUATE STUDY IN SCOTLAND AND IRELAND.

The four Scottish Universities have been but little distinguished in the past for their encouragement of post-graduate study. St. Andrews and Aberdeen concern themselves with the graduate no further than by offering him a degree in Public Health. Beyond this, Glasgow University offers courses in practical bacteriology, pathological histology, physiological chemistry, embryology, and a few of the special departments. Special courses are provided at Anderson's College Medical School and at St. Mungo's College. At the Glasgow Royal Infirmary the members of the hospital staff have now arranged an eighth series of post-graduate classes. These are to be opened on September 1. The staff of the Royal Infirmary have organised a comprehensive series of practical and clinical classes. The previous courses have already proved very successful, and have been well attended. Apply to Dr. Maxtone Thom, Superintendent of the Royal Infirmary.

In Edinburgh a more elaborate scheme of post-graduate study is available. The University of this city, in addition to teaching the subject of public health, has for some years provided advanced classes in the ordinary medical subjects for senior students and graduates, such as bacteriology, organic chemistry, and embryology, while numerous laboratories attached to the various professional chairs have been open for research under certain restrictions. These laboratories are increasing yearly in equipment and efficiency, mainly as a result of the funds derived from Mr. Andrew Carnegie's gift of £2,000,000 to the Scottish Universities. For this purpose, too, a magnificent laboratory has been organised and equipped by the Royal College of Physicians of Edinburgh, where any medical graduate may obtain permission to conduct scientific research.

The University and the Royal Colleges of Edinburgh provide courses for graduates who have obtained their diploma—medical officers home on furlough, practitioners resident in country districts, foreign doctors anxious to study English, and any others who may desire a brief *résumé* of the essentials and recent advances in various special subjects.

A three weeks' Post-Graduate Course is given during the autumn session (September 25 to October 14) at Trinity College, Dublin. The fee is £5 5s. A limited number of the class can live in the College rooms and dine in hall, at a charge of £1 per week. Apply Dr. Alfred Parsons, 27 Lower Fitzwilliam Street, Dublin.

